# Hospitalizations due injuries resultant from ground traffic accidents in Brazil from 2008 to 2016

**Published:** 2019-08

**Authors:** Érika Carvalho de Aquino, Vinícius da Silva Oliveira, Everaldo Correia de Lima Júnior, Otaliba Libânio de Morais Neto

**Affiliations:** ^*a*^Universidade Fernando Pessoa, Brazil.; ^*b*^Universidade Federal de Goiás, Brazil.

## Abstract

**Background:**

In 2015, data from low and middle income countries, victims of land transportation accidents (LTA) accounted for 13 to 31% of hospital visits due to external causes, and 48% of beds in surgical wards. In addition, these individuals required more frequent attention in intensive care units, radiology and physiotherapy or rehabilitation services. In 2016, there were 337.190 LTA victims in Brazil, 94.4% of them non-fatal. Regarding the relevance of this issue, the objective of the authors is to analyze the trend of hospitalization rates by LTA in Brazilian capitals, in the period from 2008 to 2016.

**Methods:**

Data concerning hospitalizations were obtained from the Hospital Information System. Those with basic cause V01 to V89 of the ICD-10 were considered deaths by LTA. Annual IBGE population estimates was used. These data were collected from the website of the Department of Informatics of the National Health System. The specific annual rates of hospitalization for LTA were standardized by age. The Prais-Winsten method of linear regression was used to estimate the trends. P = 0.05 was used as the critical value.The analyzes were performed using the STATA 14.0 software.

**Results:**

In 2008 there were 14.2 admissions / 100 thousand people in Brazil. In 2016, 16.6 hospitalizations / 100 thousand people (17%). In 2008, the highest hospitalization rate was observed among pedestrians (5.5 hospitalizations / 100 thousand people). As early as 2016, the highest rate was noticed among motorcycle drivers (9.7 / 100,000 people). The highest percentage change was observed in the city of Palmas (from 2.9 / 100 thousand to 166.5 / 100 thousand). There was a trend of increased hospitalization rates, mainly among pedestrians and motorcycle occupants in the North and Northeast regions of the country.

**Conclusions:**

The significant impact of LTA morbidity on the Brazilian population is one of the greatest challenges for public managers today. The results of the present study may guide more effective public policies in controlling this situation.

**Keywords:**

Hospitalizations, Traffic Injuries, Brazil

